# Computational Characterization of the Role of LEM2/LaminA Interactions on the Stability of BAF‐Dimer Using Molecular Simulations

**DOI:** 10.1002/prot.70105

**Published:** 2025-12-18

**Authors:** Aswin Vinod Muthachikavil, Alexander von Appen, Thomas D. Kühne

**Affiliations:** ^1^ Center for Advanced Systems Understanding (CASUS), Helmholtz‐Zentrum Dresden‐Rossendorf Görlitz Germany; ^2^ Max Planck Institute for Cell Biology and Genetics Dresden Germany

**Keywords:** BAF, barrier‐to‐auto‐integration factor, free energy of binding, MARTINI, molecular dynamics, potential of mean force

## Abstract

The effect of the presence of the BAF‐binding LEM‐domain and LaminA Ig‐fold on the stability of the BAF dimer was studied qualitatively using non‐equilibrium pull simulations and quantitatively through the calculation of the potential of mean force profile along BAF–BAF separation distance. We find that hydrophobicity plays a significant role in stabilizing the BAF dimer when LEM‐domain and LaminA are bound. The role of LEM‐domain and LaminA in stabilizing the BAF dimer is explored by quantifying the strength of interaction between them, which are critical components of the nuclear lamina.

## 1 Introduction

The barrier‐to‐autointegration factor (BAF) protein plays an important role in organizing DNA at the inner nuclear membrane of the nuclear envelope [1, 2, 3, 4, 5]. BAF is an abundant (about 4 μM in the cell [6, 7]) and highly conserved protein that binds to double‐stranded DNA (dsDNA), lamina proteins, and other transcription factors [3]. The protein was named based on its ability to prevent the autointegration of the Moloney Murine Leukemia virus [8].

BAF forms a stable homodimer, and each constituent monomer can bind to double‐stranded DNA, independent of the sequence of the DNA [2]. The binding of this protein onto DNA is perceived to be through the contacts between the amide groups on the protein's backbone and the DNA phosphates [8, 9]. With this ability to stably bind with DNA, BAF plays a major role in the “cross‐bridging” of DNA strands, which is required to establish functional nuclear morphology following open vertebrate mitosis and for nuclear envelope rupture [10, 11, 12]. BAF also interacts with the LAP2‐Emerin‐MAN1 (LEM) domain proteins [13, 14, 15, 16]. A‐type Lamins also interact with BAF (through a different interface) and help in the formation of lamina in metazoan cells. BAF acts as a bridge between the LEM‐domain proteins and the A‐type Lamin, thereby stabilizing the LEM–Lamin connection [17]. Mutations at the interface between LaminA/C and BAF cause progeroid syndromes [17].

The formation of a BAF–LEM–LaminA complex indicates that the BAF, A‐type lamins, and the LEM‐domain proteins work synergistically [13]. Molecular dynamics simulations are an effective tool to explore the underlying synergy between these proteins. Shang et al. [18] investigated the interaction between the BAF protein (and its mutant) with DNA using molecular simulations. They provided a qualitative description of the strength of interaction between BAF and DNA. In a later work, they also showed the interaction between the BAF protein and the LEM‐domain of emerin [19]. A more recent study by Marcelot et al. studied the BAF‐dimer using all‐atom molecular simulations. They studied the effect of phosphorylation of the N‐terminus of BAF molecules and showed that the confirmation of the N‐terminal region of BAF is highly tunable based on the ionic strength and pH [2].

Despite the importance of the BAF–LEM–LaminA assembly and the wealth of literature exploring these proteins, the stability of the BAF–LEM–LaminA assembly and the role of individual proteins on its stability are not well reported in the literature. In this work, we use nonequilibrium force–probe simulations to quantify the strength of interaction between these proteins. All‐atom simulations are compared with the MARTINI 3 coarse‐graining approach. Further, the free energy profiles of interaction between these proteins are estimated using umbrella sampling simulations. The study sheds light on the synergy between these proteins, as integral components of the nuclear lamina.

## 2 Methods

### 2.1 Initial Protein Configurations

The structural model for LEM2's LEM‐domain (1Met–71Asp) was generated by AlphaFold [20] and aligned to the Emerin–LEM‐domain in complex with the BAF dimer (2Thr–89Leu) bound to the immunoglobulin‐like domain LaminA (428Ser–546Ser) domain (BAF–LEM (Emerin)–LaminA [17], PDB 6GHD) using ChimeraX [21, 22, 23] match maker.

### 2.2 All Atom Simulations

All atom simulations were performed using the CHARMM 36 m force‐field [24]. The protein assemblies of interest were placed in the center of the box, and water molecules were inserted in the box randomly, ensuring no overlaps and that the density was close to the experimental density of water. The TIP3P water model [25] was used to represent water molecules. K^+^ and Cl^−^ ions were also inserted into the simulation box to generate a neutral system with a salt concentration of 0.15 mM. The BAF dimer system created this way had two BAF molecules, 246 K^+^ particles, 244 Cl^−^ particles, and 85 388 TIP3P water molecules. The system had 259 430 particles in a cubic simulation box, approximately 14 nm long. Energy minimization of the system was performed using the steepest descent algorithm. Subsequently, the system's temperature was equilibrated at 298.15 K with 5 ns long NVT simulation using the Nose‐Hoover algorithm [26, 27]. The time constant for temperature coupling was 1 ps. The system's pressure was then equilibrated with 5 ns long NPT simulation. Berendsen barostat [28] was used to maintain pressure at 1 bar, using a time constant of 5.0 ps. During the energy‐minimization and equilibration simulations, position restraints were applied to the heavy atoms of the proteins. During production simulations, the temperature and pressure of the system were maintained using the Nose–Hoover thermostat [26, 27] and the isotropic Parrinello–Rahmann barostat [29], with coupling times of 1.0 and 2.0 ps, respectively. In all of the all‐atom simulations, long‐range electrostatic contributions were estimated using the Particle Mesh Ewald method [30, 31], with a cut‐off distance of 1.4 nm. Van der Waals interactions were also cut off at 1.4 nm, and long‐range dispersion corrections were applied. Time integration was performed using the Leap‐frog algorithm [32], with a time‐step of 1 fs.

In pull‐simulation, two groups were pulled away from each other using harmonic springs of spring constant 1000 kJ mol^−1^ nm^−2^. Unless otherwise mentioned, the groups were pulled away at a speed of 0.025 m s^−1^. In all‐atom BAF–BAF pull simulations, position restraints were applied to the heavy atoms of one BAF molecule when the other BAF molecule was pulled away. Umbrella sampling [33] simulation using CHARMM‐36 m force‐field was only performed for the BAF‐only system. The window size was maintained to be around 10 Å to ensure sufficient sampling. A harmonic spring of spring constant 1000 kJ mol^−1^ nm^−2^ was used to attach the proteins to the center of each sampling window to ensure sampling inside the specific window. A higher spring constant (3500 kJ mol^−1^ nm^−2^) was also used for sampling windows close to free‐energy minimum. 150 ns long simulations were used to generate histograms, followed by Weighted Histogram Analysis Method (WHAM) [34] (implemented in Gromacs [35]) to estimate the PMF profile.

### 2.3 Coarse‐Grained Simulations

Coarse‐grained simulations were performed using the MARTINI 3 force‐field [36]. The atomistic molecular structures of the proteins were converted to MARTINI 3 coarse‐grained structures using the Martinize 2 script [37]. Nonpolarizable water beads and ions were inserted into the simulation box using the “insane” (INSert membrANE) python script [38], such that the system's density was close to that of water at standard conditions, and the salt concentration was 0.15 mM. The system created this way had the protein molecules, ions, and nonpolarizable water beads. The size of the simulation box was determined such that the length of the simulation box was at least twice the size of the specific protein assembly. The systems created this way were energy‐minimized using the conjugate gradient algorithm [39, 40]. The temperature of the system was equilibrated at 298.15 K with 5 ns long NVT simulations, using the V‐rescale thermostat [41]. Subsequently, the system's pressure was equilibrated with 5 ns NPT simulations, using the Berendsen barostat [28]. Position restraints were applied to the atoms of the protein molecules while performing equilibration simulations. In production simulations, temperature and pressure were maintained at 195.1 K and 1 bar using the Nose‐Hoover thermostat [26, 27] and the isotropic Parrinello–Rahmann barostat [29], respectively. Standard cut‐off schemes are used for nonbonded interactions, as these are part of the MARTINI parametrization. Dispersion interactions are shifted to zero at a distance of 1.1 nm. Electrostatic interactions were calculated using a reaction field. Beyond the cut‐off of 1.1 nm, an infinite dielectric constant was assumed. A time‐step of 10 fs was used to perform time integration using the Leapfrog algorithm. The lengths of equilibrium production simulations were decided based on the convergence of macroscopic thermodynamic properties and the reported observables of the system.

In force–probe simulations (or pull‐simulations) where one group was pulled away from another reference group, position restraints were applied to the atoms of all other proteins other than the one that was being pulled. Pulling was done by a harmonic spring of spring constant 1000 kJ mol^−1^ nm^−2^. The velocity of pulling was 0.025 m s^−1^, unless otherwise specified in relevant portions of the article.

Umbrella sampling [33] was used to estimate the potential of mean force (PMF) profiles along the distance of separation between two proteins of interest. The maximum width of umbrella sampling windows was about 10 Å to ensure sufficient sampling along the reaction coordinate (separation distance between two proteins). A harmonic spring of spring constant 1000 kJ mol^−1^ nm^−2^ was used to attach the proteins to the center of each sampling window to ensure sampling inside the specific window. Higher spring constants (2500 or 3500 kJ mol^−1^ nm^−2^) were also used in some windows where it was necessary to hold the proteins within specific sampling windows, especially while sampling windows close to a very deep free energy well. The spring constants used ensured that there was significant overlap between histograms. Histograms obtained from umbrella sampling simulations were used to construct the PMF profiles using WHAM [34] implemented by Gromacs [35]. All molecular dynamics simulations were performed using Gromacs 2023.2 [42, 43]. We note that in both pulling and umbrella sampling simulations, position restraints are applied to all backbone atoms of one BAF monomer. This approach may suppress the conformational flexibility of the restrained monomer during rupture.

## 3 Results and Discussion

### 3.1 Stability of the BAF Dimer

Coarse‐graining is often advantageous in molecular simulations to reduce computational costs, although at the expense of the accuracy of the underlying potential energy surfaces (PES). Coarse‐graining helps to overcome the bottlenecks of length and time scales in classical all‐atom molecular dynamics simulations [36]. These are especially beneficial in biomolecular simulations where the system sizes and length scales to be probed are generally larger. The MARTINI coarse‐grained force‐field is widely used in many areas of research including structural biology [44, 45, 46], biophysics [47, 48], biomedicine [49], and many others [50, 51, 52, 53].

**FIGURE 1:**
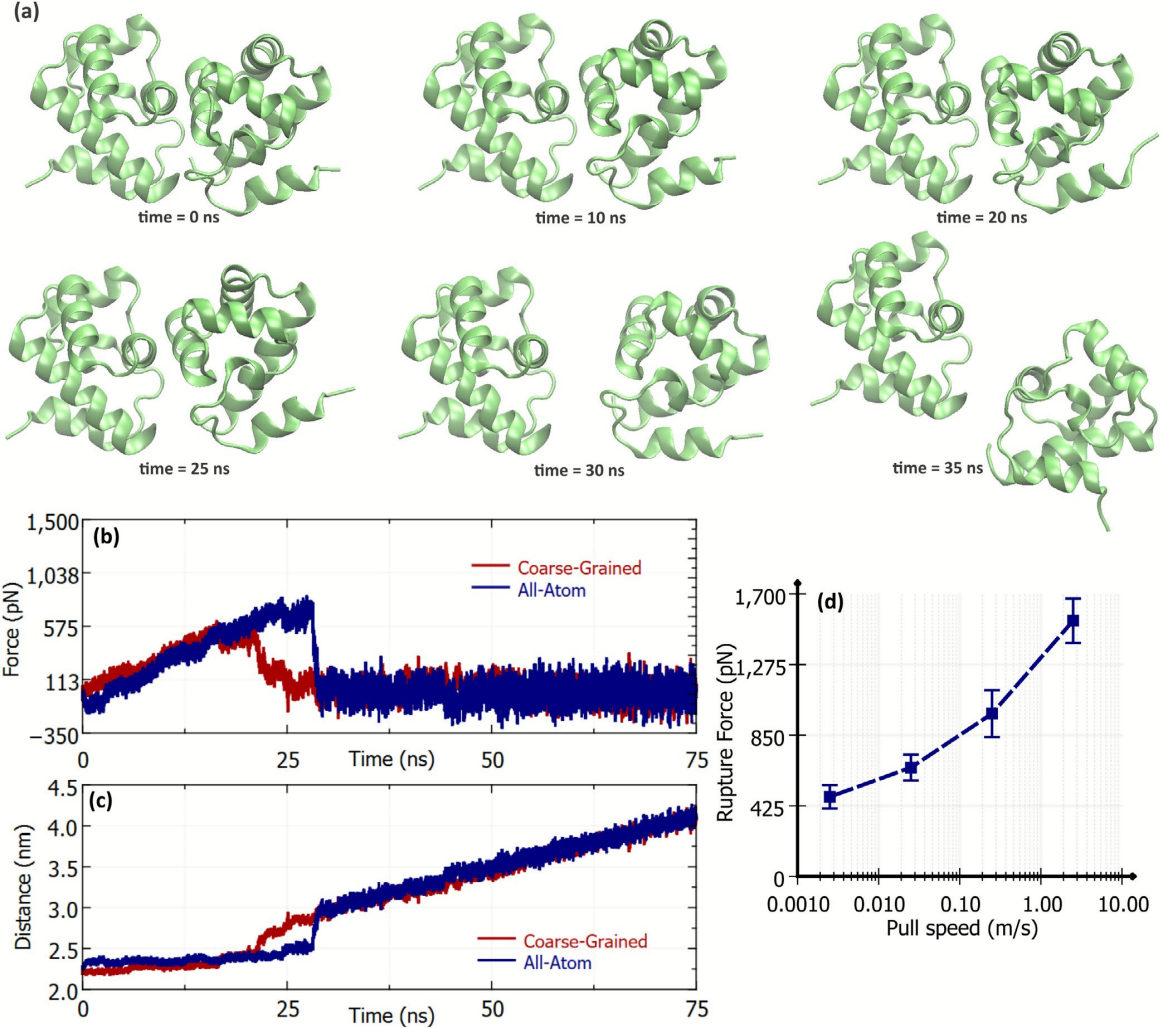
(a) Visualization of pull simulations where a BAF molecule is pulled from its pair with a harmonic spring at a constant pull velocity of 0.025 nm s^−1^. One BAF monomer is pulled away while position restraints are applied to the backbone atoms of the other. The visuals are from simulations using the CHARMM 36 m all‐atom force‐field. Because of the affinity of the BAF molecule to exist in a dimerized state, the pulled protein remains bound to its pair until the force on the spring is strong enough to rupture the dimer. (b and c) Comparisons of pull simulations using the CHARMM 36 m force‐field (all‐atom) and MARTINI 3 (coarse‐grained) simulations of the BAF dimer. (b) The variation in force on the spring, as one BAF molecule is pulled away from its pair, and (b) the distance between the center of mass (COM) of the proteins as they are pulled apart. (d) The variation of rupture force with pull speed for the BAF dimers. Rupture force is estimated as the maximum force on the harmonic spring when the two monomers disassociate from each other. The error bars in (d) represent the standard deviation of the rupture force obtained from 8 independent pull‐simulations.

Since coarse‐graining leads to a loss of resolution in the underlying PES of the system, we investigated if this loss of resolution results in qualitatively different behaviors between the coarse‐grained and all‐atom simulations. We compared the results between an all‐atom and coarse‐grained simulations when a BAF molecule is pulled away from its pair. There is an inherent affinity of the BAF molecules to exist in a dimerized form [2]. Therefore, when one molecule is pulled away from its pair with a harmonic spring at a constant velocity, the other BAF molecule tries to hold its pair in position. This leads to a linear increase in the force on the spring until the BAF dimer ruptures and the monomers are separated from each other. Visualization of the trajectory of such a “pull simulation” is shown in Figure 1a. The force on the spring and the distance between two BAF molecules in such a simulation are shown in Figure 1 (b and c, respectively). The red lines represent simulations using MARTINI 3 force‐field, and the blue lines represent simulations using the CHARMM36m force field. We observe that the force–time characteristics of all‐atom simulations are qualitatively reproduced in the coarse‐grained simulations using the MARTINI 3 force‐field. The magnitude of the forces and the nature of the rupture are fairly well captured in MARTINI 3 simulations. However, a general “loss of sharpness” can be observed in both the force‐time and distance–time curves of coarse‐grained simulations. We believe that this difference is because of the loss in resolution of the PES inherent to the coarse‐graining process. Given the computational advantages of coarse‐graining and the fact that the qualitative behavior of the system is not lost, the MARTINI force field is used to produce the results reported in the rest of the article.

The speed of pulling is an important parameter in such force–probe simulations. While experimental pulling techniques like force spectroscopy use very small pulling speeds (10^−5^ to 10^−8^ m s^−1^), simulations usually employ higher pulling speeds of roughly 10^−3^ to 1 m s^−1^ owing to computational restrictions [54]. In studies involving the probing of protein‐unfolding, the order of unfolding can differ with the rate of pulling, because the molecule can cross many free energy barriers on the multidimensional free energy landscapes of the system, depending on the pulling rate [55, 56, 57, 58]. According to microscopic theories describing the kinetics of rupture, the average force at rupture is a nonlinear function of the logarithm of the pulling speed [59]. Specifically, there is an increase in the rupture force with the speed of pulling [59] (see Figure 1d). It is beneficial to use smaller pulling speeds if the simulation aims to test the quantitative reproducibility of experiments. However, the time required to attain a rupture event is also longer at slower pulling speeds. The computational expense associated with such long simulations is a bottleneck to studying larger and varied systems. In further sections of the article, we discuss the stability of the BAF dimer in the presence of other protein molecules using force probe simulations. In these systems, we use a pulling speed of 2.5 × 10–2 m s^−1^ to study the binding affinity of BAF molecules in the presence of other proteins.

#### 3.1.1 The Role of LEM‐Domain and LaminA

**FIGURE 2:**
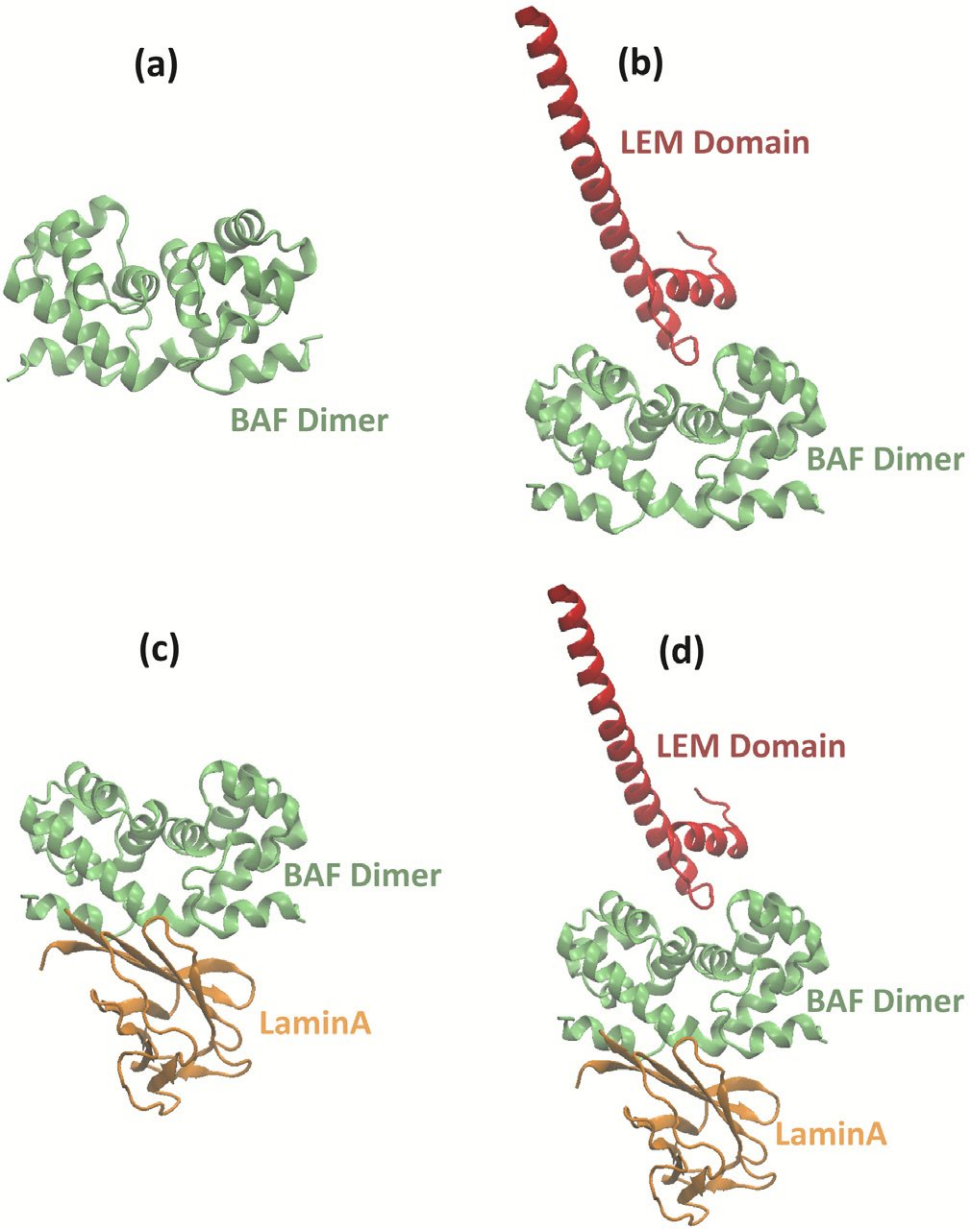
Visualization of BAF dimer with other proteins (LaminA and the LEM‐domain of the LAP2) that often appear with it. To study the effect of these proteins on the stability of the BAF dimer, we conducted simulations on a BAF dimer (a) in the absence of any other proteins (b) in the presence of the LEM‐domain of the LAP2 protein (c) in the presence of LaminA (d) in the presence of both the LEM‐domain and LaminA. In each of these systems, the stability of the BAF dimer was estimated and later compared.

LaminA and the LEM‐domain of the LAP2 protein are two proteins that bind to the BAF protein and work in tandem with it, playing a role in bridging DNA during cell division. To understand the interactions between these proteins and to quantify the effect of the LEM‐domain and LaminA on the stability of the BAF dimer, different systems of protein assemblies were studied in this work (shown in Figure 2). The stability of the BAF dimer in each of these systems was studied separately and compared.

**FIGURE 3:**
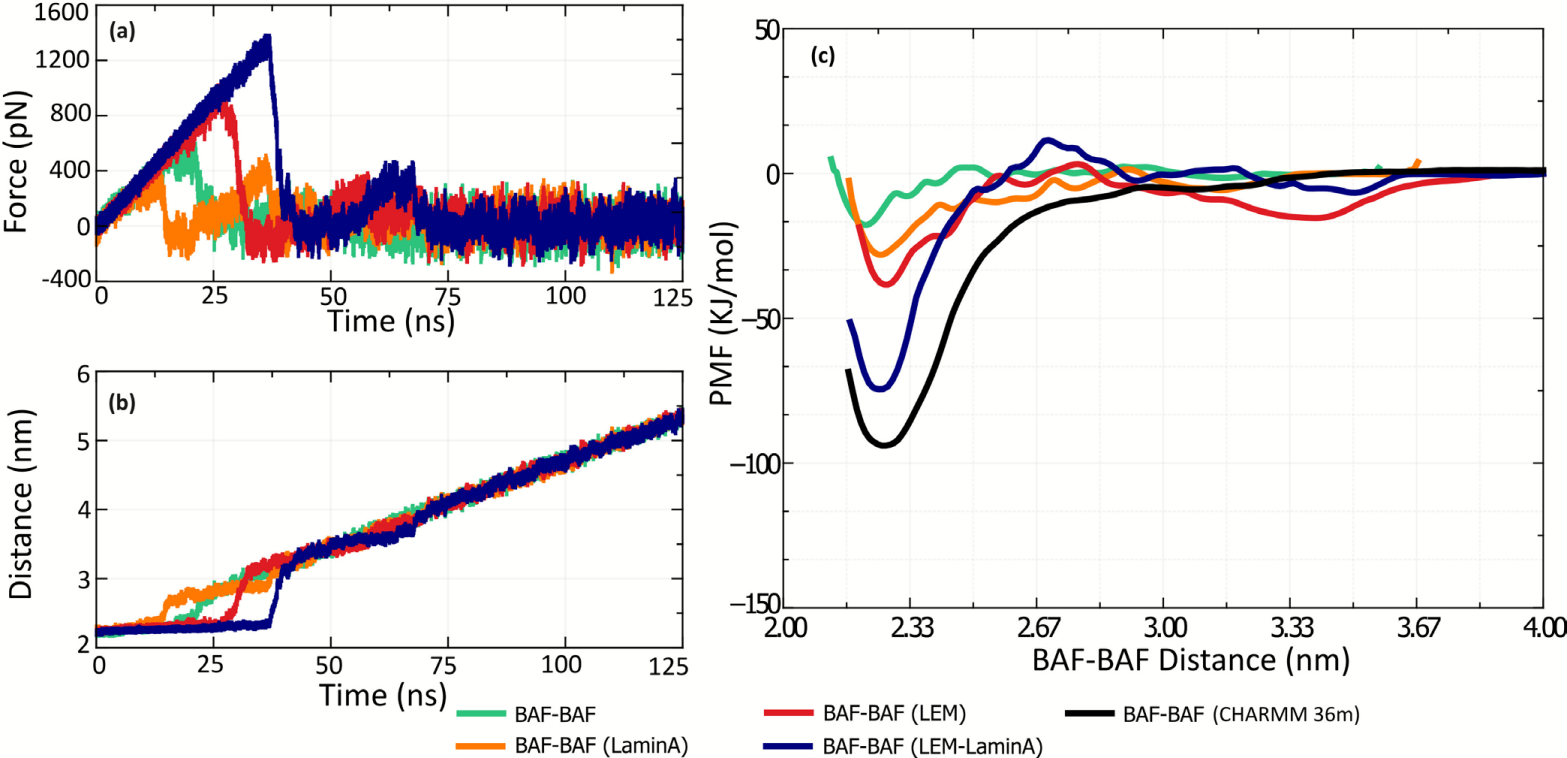
(a) Pull force when a BAF monomer is pulled away from its pair in a dimer configuration in the presence of other proteins (LaminA and the LEM‐domain of the LAP2 protein). (b) Corresponding distance between the COM of the two BAF monomers as they are pulled apart with a harmonic spring (*k* = 1000 kJ nm^−1^ mol^−1^) at a constant velocity (*v* = 0.0025 m s^−1^). (c) Potential of mean force (PMF) profile along BAF–BAF separation distance for different systems studied. The green line represents the system with no other proteins other than BAF. The orange line represents the system when only LaminA is present. The red line represents the system where only the LEM‐domain is present, bound to the BAF dimer. The blue line represents the system where both LEM and LaminA are present, bound to the BAF dimer. The results shown are from MARTINI simulations. To compare, we also show the PMF profile of BAF‐only system using the CHARMM‐36 m all‐atom force‐field (shown in black).

As discussed earlier in the article, a qualitative understanding of the stability of the BAF dimer can be obtained from force–probe simulations. Figure 3a,b shows the force and distance–time characteristics of BAF‐BAF pull simulations in the presence of LEM and/or LaminA. The force required to rupture the BAF dimer is larger for the BAF–LEM and the BAF–LEM–LaminA systems, compared to a BAF‐only system (see Figure 3a). In these systems, the rupture happens in a single “snap,” as the separation between the monomers changes from 2.3 to 3 nm. This is indicated by the single “peak” in the force–time characteristics (when the distance is between 2.3 and 3.0 nm). On the contrary, the rupture of the BAF dimer in the BAF–LaminA system is observed to happen in two distinct “snaps” as the BAF monomers are separated between 2.3 and 3 nm. The magnitude of force at both these snaps is lower than that of the BAF‐only simulations. This observation points to the possibility that the presence of LaminA alone reduces the stability of the BAF dimer. However, before making this conclusion, one should note the presence of two peaks in the force–time characteristics of the BAF‐LaminA system, within a short range of distance (2.3–3 nm) between the monomers, before the rupture of the dimer. These results also depend on parameters that are external to the system, like the stiffness of the harmonic spring and the velocity of pulling (see Figure 1d for example). Therefore, to comment conclusively on the stability of the BAF dimer in the presence of the LEM‐domain and LaminA, we computed the PMF profile along the distance of separation between the BAF monomers. It is to be noted that the reaction coordinate is not a specific direction (along *X*‐, *Y*‐, *Z*‐axes, or any other direction relative to the interface of the protein), but the distance of separation between the BAF monomers. The PMF profile gives us the free energy landscape along the separation distance between BAF monomers, which is solely dependent on the system under investigation, and not on external variables that are involved in nonequilibrium simulations.

The PMF profiles along BAF–BAF separation distance for all the systems studied are shown in Figure 3c. The “free energy well” at BAF‐BAF separation of about 2.3 nm points to an equilibrium binding distance between the monomers. The depth of the free energy well is a direct indicator of the stability. The PMF profile indicates that the stability of the BAF dimer increases when LaminA and the LEM‐domain are bound to the dimer. Specifically, the deepest PMF well (−75 kJ/mol) is observed for the BAF–LEM–LaminA system. Although both LaminA and LEM‐domain are individually observed to stabilize the BAF dimer, their presence together has a much higher impact on the stability of the BAF dimer. We also computed the all‐atom PMF profiles for the BAF‐only system (black line in Figure 3c). Although qualitatively similar to that of the MARTINI 3 results, the PMF well is observed to be deeper in all‐atom simulations. Such deviations between free‐energy profiles between all‐atom and coarse‐grained force‐fields are reported in the literature [60, 61, 62, 63, 64, 65]. MARTINI 3 has been reported specifically to underestimate the free‐energies of binding in simulations, while MARTINI 2 has been reported to overestimate them [65]. The difference in the depth of free‐energy wells potentially arises from the deviation in enthalpy/entropy decomposition in coarse‐grained models, and the resultant temperature‐transferability challenges that are inherent to MARTINI [61]. However, even though the force–field underpredicts the free energy of binding, the qualitative trends are often reproduced well by MARTINI 3 [65]. Owing to reduced computational expenses, they enable extensive sampling while maintaining the general qualitative behavior of the system [66]. This agrees with our results, since the qualitative behavior of BAF‐binding is not lost in coarse‐grained simulations, as indicated by pulling simulations and the BAF‐BAF PMF profile.

### 3.2 Structural Insights

**FIGURE 4:**
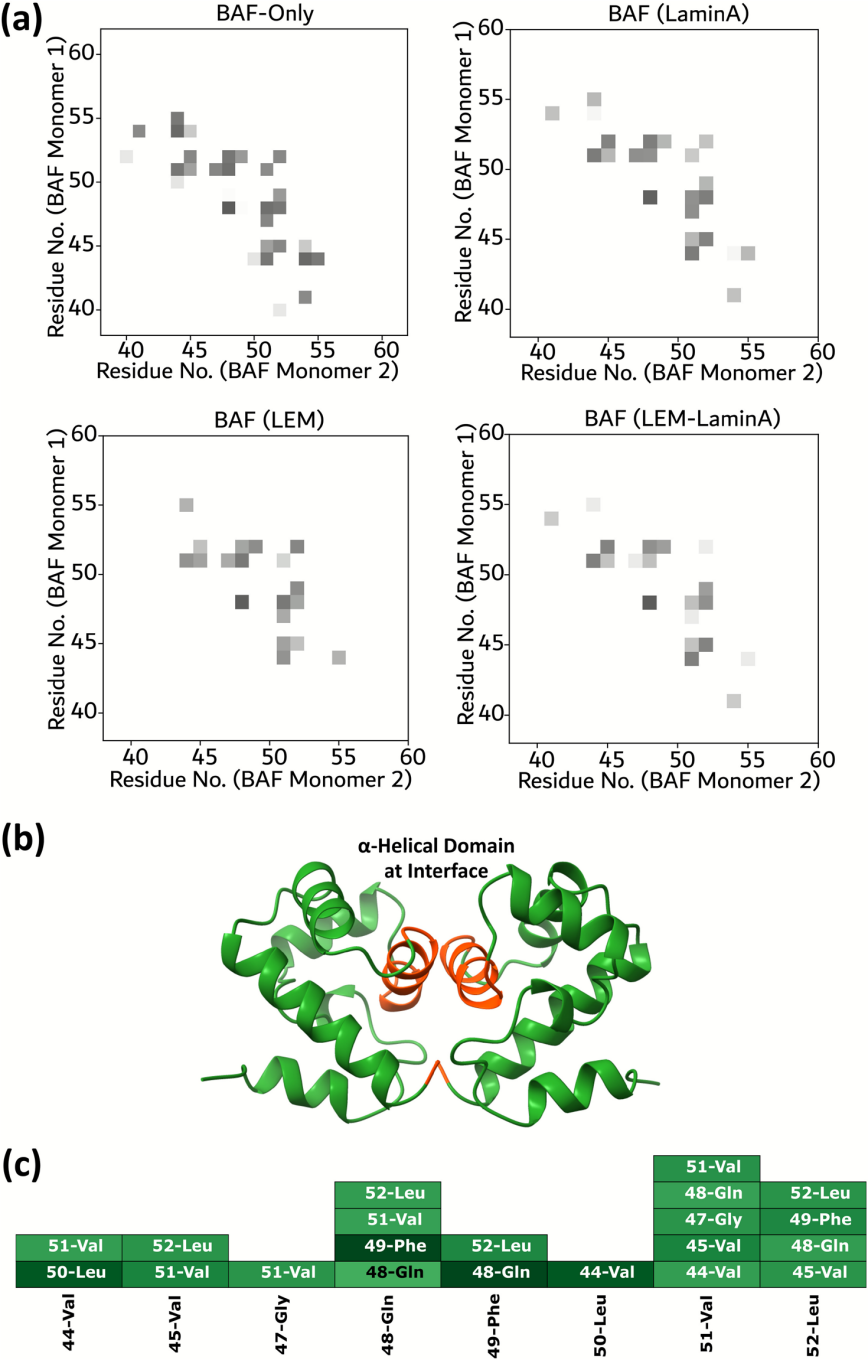
(a) Contact map between two BAF monomers. The contact‐map shows the interaction between residues that are around the α‐helical domain at the interface. The points in the figure indicate the residues that make contact between the two monomers. The darker the color, the closer the residues are to each other. Results are shown for (i) BAF‐only, (ii), BAF–LaminA, (iii) BAF–LEM, and (iv) BAF–LEM–LaminA systems. (b) In a dimerized state, the contacts between BAF monomers in a dimerized state happen mainly through the α‐helical domain (shown in red). (c) The important contacts between the α‐helical domains of BAF monomers. Darker colors indicate closer contacts.

The results discussed so far indicate that there is an increase in the stability of the BAF dimer when the LEM‐domain and LaminA are bound to it. In the current section, we provide insights into the molecular origin of this increase in stability due to the presence of the LEM‐domain and LaminA. Atomic contacts between two molecules often play an important role in the stabilization of intermolecular complexes. The important contacts between two BAF monomers obtained from equilibrium simulations are shown in Figure 4. Two residues were defined to be in “contact” if the minimum distance between the atoms of the two residues was less than 6 Å.

**FIGURE 5:**
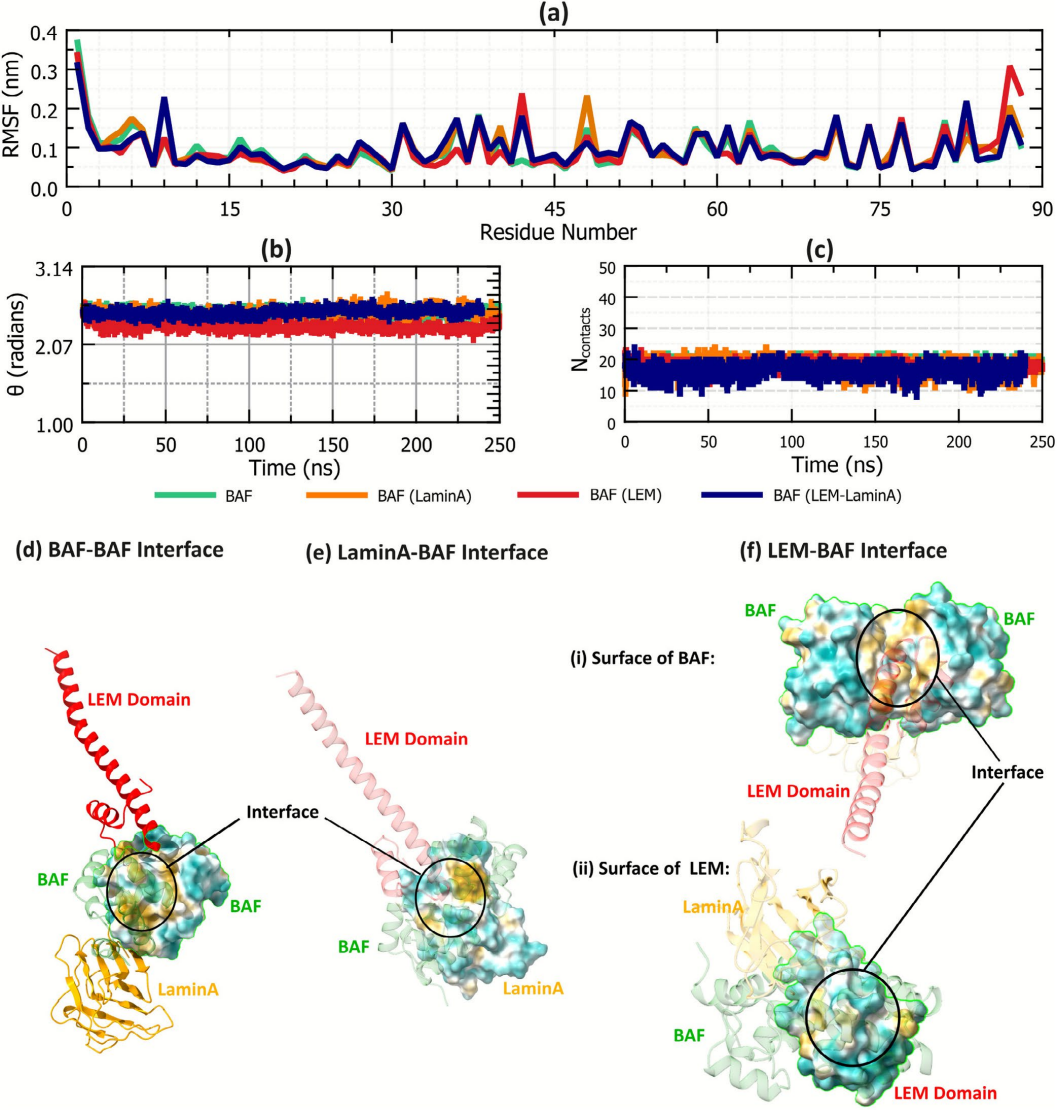
(a) Root mean squared fluctuations of the residues of BAF monomers in equilibrium simulations. (b) The mutual orientation between the interacting surface of the BAF monomers, measured as the angle between the axes of the α‐helices at the interface. (c) Number of contacts between two BAF monomers plotted against time. The contacts made between the α‐helical domains at the BAF–BAF interface (LYS 41–LEU 52 as shown in Figure 4b) are shown here. (d–f) Hydrophobicity of the surfaces of interaction within the BAF–LEM–LaminA complex at the (d) BAF–BAF interface, (e) LaminA–BAF interface, and (f) LEM–BAF interface. Hydrophobicity of residues are according to the definition provided by Kyte et al. [67]. Brown color indicates hydrophobic residues and blue color indicates hydrophilic residues. Visualization is using ChimeraX [21, 22, 23].

The contact map between the residues of BAF is shown in Figure 4a. In all the configurations studied (BAF only, BAF–LEM, BAF–LaminA, and BAF–LEM–LaminA), contacts between two BAF monomers occur mainly through the predominantly α‐helical domain at the interface (LYS 41–LEU 52, shown in Figure 4b). The similarity of BAF–BAF interaction in all the systems studied is also indicated by a fairly constant number of contacts made through the α‐helical region at the interface (as shown later in Figure 5c). Most of these interactions are between hydrophobic residues (44Val–50Leu, 44Val–51Val, 45Val–51Val, 45Val–52Leu). The importance of these hydrophobic residues in stabilizing the BAF assembly is discussed later in Section 3.2.1. Other residues involved in the contact between BAF monomers are 47Gly, 48Gln (Polar), and 49Phe (Aromatic). Figure 4c shows a color‐coded list of contacts between the α‐helical domains of the monomers.

To explore the effect of the presence of LEM and LaminA on the structure of the BAF–dimer assembly, the structural fluctuations and the mutual orientation between the monomers were also measured, along with the contacts between the monomers. The root mean‐squared fluctuations (RMSF) of each BAF residue in equilibrium simulations are shown in Figure 5a. In general, the residues at both the coiled tail ends of the BAF protein have more structural fluctuations. However, there is no significant difference between the RMSF of BAF in BAF‐only, BAF–LaminA, BAF–LEM, and BAF–LEM–LaminA systems, and hence it is inferred not to be a significant contributing factor in the stability of the dimer.

The mutual orientation is also often important in forming stable intermolecular complexes. In this work, the mutual orientation between the interacting α‐helical regions was quantified by calculating the angle between the unit vectors representing the axes of the helices. The angle (*θ*) between the helices was calculated as $\cos^{-1}\left(\vec{h_{1}}\cdot \vec{h_{2}}\right)$, where $\vec{h_{1}}$and $\vec{h_{2}}$ are the unit vectors along the axis of the α‐helices at the interface (shown in Figure 4b) of BAF monomers 1 and monomer 2, respectively. The variation of *θ* with time is shown in Figure 5b. The value of *θ* is observed to be stable throughout the simulation trajectory, presumably because of the stability of the BAF–BAF conformation in the dimer. Interestingly, the presence of LaminA or LEM‐domain was observed not to significantly impact the mutual orientation of BAF monomers. In BAF‐only, BAF–LaminA, BAF–LEM, and BAF–LEM–LaminA systems, there is no significant deviation in the values of *θ*. Therefore, the presence of LEM and/or LaminA does not have an impact on the contacts, and the mutual orientation between the BAF dimer, but plays an important role in the stability as indicated by the free‐energy of binding.

#### 3.2.1 Hydrophobicity

All the results discussed so far quantify the structure, the contacts, and the orientation between two BAF monomers in their dimerized state. The change in the orientation and contacts between two molecules implies a change in the potential energy of the system. A variation in the number of contacts and/or orientation with each other in the presence of LEM and LaminA would imply that the energy of the system has a determining role in the stabilization of the dimer. However, evidence from simulations presented so far indicate that despite an increase in the binding strength of the BAF dimer when LEM‐domain and LaminA are bound to it, there are no significant changes in the contacts between the BAF monomers. The results point to the possibility that hydrophobicity may play an important role in the change in the free energy of binding of the BAF dimer (Figure 3c). To gain further insights into the stability of the BAF dimer, we analyzed the hydrophobicity of the residues at the surface of BAF, LEM‐domain, and LaminA. The hydrophobicity of each residue as defined by Kyte et al. [67] is visualized in Figure 5d–f. Blue color is used to represent hydrophilic aminoacids and brown color is used to indicate hydrophobic residues. The exposure of water molecules to hydrophobic entities is thermodynamically unfavorable, and therefore, the free energy change associated with the rupture of an intermolecular assembly depends on the extent of hydrophobic surface that gets exposed when the assembly is ruptured [68].

Figure 5d shows the hydrophobicity of the residues at the BAF–BAF interface. The BAF–BAF interface is predominantly composed of hydrophobic residues (shown in Figure 5a, and the important BAF–BAF contacts listed in Figure 4c). Therefore, the contact of these residues with water is reduced upon dimerization of the BAF monomers.

When only LaminA is present (bound to the BAF dimer), the dimer is more stable compared to a BAF‐only system. This has been discussed previously, and is demonstrated by the PMF profiles shown in Figure 3c. The hydrophobicity of the β‐sheet surface of the LaminA interacting with the BAF dimer is shown in Figure 5e. The surface is amphiphilic, with 12 out of 33 residues (1 SER–10 SER and 97 ASN–119 SER; where the numbers represent a 1‐based indexing of the LaminA sequence studied) that interact closely with the BAF dimer being hydrophobic. When BAF monomers are separated from each other when LaminA is bound to them, both (i) the hydrophobic surface of BAF monomers and (ii) the amphiphilic surface of LaminA get exposed to water. Therefore, the combined effects from the exposure of the hydrophobic surface of BAF (at the BAF–BAF interface) and the exposure of the amphiphilic surface of LaminA (at the LaminA–BAF interface) upon rupture of the BAF dimer make the BAF dimer stronger in the presence of LaminA (compared to the BAF‐only system). Readers should note that the surface of the BAF proteins that bind with LaminA also gets exposed to solvents upon separating the BAF molecules in the presence of LaminA. However, this surface is not predominantly hydrophobic.

The surface of the LEM‐domain that binds to BAF is also amphiphilic. However, the surface of the region of BAF proteins which bind to the LEM‐domain is predominantly hydrophobic as shown in Figure 5f. Therefore, when the BAF monomers (in the presence of LEM‐domain, as shown in Figure 2b) are separated from each other, (i) the amphiphilic surface on the LEM‐domain (at LEM–BAF interface), (ii) the hydrophobic surface on the BAF dimer (at BAF–LEM interface), and (iii) the hydrophobic surface of BAF monomers (at the BAF–BAF interface) get exposed to the surrounding water. The extent of exposure of hydrophobic residues upon the rupture of the BAF dimer in the presence of an LEM‐domain is, therefore, higher than when the dimer is ruptured in a BAF‐only and BAF‐LaminA system. This results in a deeper PMF well (or a more stable BAF dimer), as shown in Figure 3c.

The presence of both LEM and LaminA combines the effects of both. When separating two BAF dimers from each other in the presence of both LEM‐domain and LaminA, there is an exposure of the surfaces that would have been exposed when (i) only LaminA is present and (ii) only LEM is present. Therefore, the BAF dimer is the most stable when both LEM‐domain and LaminA are bound to it, as shown in Figure 3c. A near‐linear dependence is observed between the free energies of binding of BAF monomers in BAF–LaminA, BAF–LEM, and BAF–LEM–LaminA configurations. That is, the free energy of binding of BAF monomers in the BAF–LEM–LaminA (∼74 kJ mol^−1^) is close to the sum of the BAF–LEM (∼38.75 kJ mol^−1^) and BAF–LaminA (∼28.75 kJ mol^−1^) configurations.

The analysis of the hydrophobicity of the surfaces of interaction within the BAF–LEM–LaminA complex indicates that the stability of the BAF dimer can be attributed to the exposure of hydrophobic surfaces upon its rupture, and that the increased stability of the BAF dimer in the presence of LEM‐domain and LaminA is predominantly because of hydrophobic effects.

## 4 Conclusions

The role of the LEM domain of the LAP2 protein and LaminA on the stability of the BAF dimer was studied using all‐atom and coarse‐grained simulations. For this purpose, the force‐time and distance‐time characteristics while pulling a BAF monomer away from its pair were studied for BAF‐only, BAF–LEM, BAF–LaminA, and BAF–LEM–LaminA systems. Further, for a quantitative understanding that does not depend on external variables involved in nonequilibrium simulations, the PMF‐profile of binding between BAF–BAF monomers was estimated for all the systems mentioned. It was observed that the presence of the LEM domain and LaminA increases the free energy of binding between the BAF dimer. The free energy of binding has both energetic and entropic contributions. To understand if the BAF dimer's stability originates from energetic or entropic contributions, we studied the effect of LEM and LaminA on the contacts between the BAF dimers and the hydrophobicity of the surface of the BAF–LEM–LaminA complex.

In the dimerized state, the contacts between BAF‐monomers are predominantly through an α‐helical domain (LYS 41–LEU 52). However, the nature and number of contacts between the dimer remain stable and are unaffected by the presence of LEM‐domain and/or LaminA. The presence of these proteins also has a very minimal effect on the mutual orientation between the two monomers in equilibrium simulations. This suggests that the energy of interaction between the two monomers is not significantly affected by the presence of LEM or LaminA, and the stability of the BAF dimer does not stem from energetic reasons.

On the other hand, an analysis of the hydrophobicity of the surface of the BAF–LEM–LaminA provides a clearer picture of the reasons behind the stability of the assembly. The unfavorable exposure of hydrophobic surfaces upon the rupture of the BAF dimer may lie at the root of the stability of the BAF–LEM–LaminA complex. This also provides a consistent explanation for the variation of the free energy of binding when the LEM‐domain and/or LaminA are bound to the BAF dimer.

The results presented in the article provide an understanding of the strength of interaction between BAF dimers and BAF monomers, and the effect of the LEM‐domain and LaminA on it. The work also probes the reasons behind the stability of the BAF–LEM–LaminA complex and points to the possibility that the stability of the dimer is primarily driven by hydrophobicity. The study also provides a useful benchmark for future research on BAF (LEM–LaminA) complex–DNA interactions.

## Author Contributions

**Aswin Vinod Muthachikavil:** investigation, methodology, writing – original draft, formal analysis, validation, writing – review and editing. **Alexander von Appen:** conceptualization, investigation, writing – review and editing, supervision, validation, formal analysis. **Thomas D. Kühne:** conceptualization, investigation, writing – review and editing, methodology, validation, supervision, formal analysis, funding acquisition.

## Data Availability

Data supporting the findings of this study are presented within the manuscript.
